# Inhibitory Antibodies Designed for Matrix Metalloproteinase Modulation

**DOI:** 10.3390/molecules24122265

**Published:** 2019-06-18

**Authors:** Thomas Fischer, Rainer Riedl

**Affiliations:** Institute of Chemistry and Biotechnology, Center of Organic and Medicinal Chemistry, Zurich University of Applied Sciences, Einsiedlerstrasse 31, 8820 Wädenswil, Switzerland; thomas.fischer@zhaw.ch

**Keywords:** inhibitory antibody, drug design, matrix metalloproteinase, tissue inhibitors of metalloproteinases, selectivity

## Abstract

The family of matrix metalloproteinases (MMPs) consists of a set of biological targets that are involved in a multitude of severe pathogenic events such as different forms of cancers or arthritis. Modulation of the target class with small molecule drugs has not led to the anticipated success until present, as all clinical trials failed due to unacceptable side effects or a lack of therapeutic outcome. Monoclonal antibodies offer a tremendous therapeutic potential given their high target selectivity and good pharmacokinetic profiles. For the treatment of a variety of diseases there are already antibody therapies available and the number is increasing. Recently, several antibodies were developed for the selective inhibition of single MMPs that showed high potency and were therefore investigated in in vivo studies with promising results. In this review, we highlight the progress that has been achieved toward the design of inhibitory antibodies that successfully modulate MMP-9 and MMP-14.

## 1. Introduction

Matrix metalloproteinases (MMPs) are zinc and calcium dependent endopeptidases that are involved in tissue remodeling and other processes where the degradation of extracellular matrix (ECM) proteins is involved. Due to their involvement in a variety of diseases, they have been of interest in pharmaceutical research for decades [1,2,3,4]. Early attempts of inhibitor development focused on strong metal chelators like hydroxamic acids that were able to block the catalytic center within the enzyme. Even though the approach delivered highly potent inhibitors, none of them survived clinical trials as a result of intolerable side effects like joint stiffening [5]. As those early inhibitors did not distinguish between individual MMPs but blocked the whole panel, further efforts were taken in the direction of finding selective inhibitors for a particular MMP family member. By moving from strong zinc binding groups, such as hydroxamic acids, to weaker chelators, such as carboxylic acids, the effect that zinc binding has on enzyme inhibition could be reduced and the impact of the remaining interactions was strengthened. As the three-dimensional structure of the active sites throughout the MMPs differ, this development yielded modulators with a more sophisticated selectivity profile than the broad-spectrum inhibitors. Further evolution targeting more selective inhibitors brought up allosteric binders that did not interact with the catalytic zinc but blocked the enzyme in a non-competitive fashion. Over time, selective inhibitors could be developed for many of the MMPs, but up to date none of them have reached the market. All of them failed in clinical trials for different reasons such as side effects or lack of the therapeutic effect. Those problems with small molecule inhibitors required new approaches to be taken in the challenging field of finding a drug that targets this intriguing class of enzymes.

**Figure 1:**
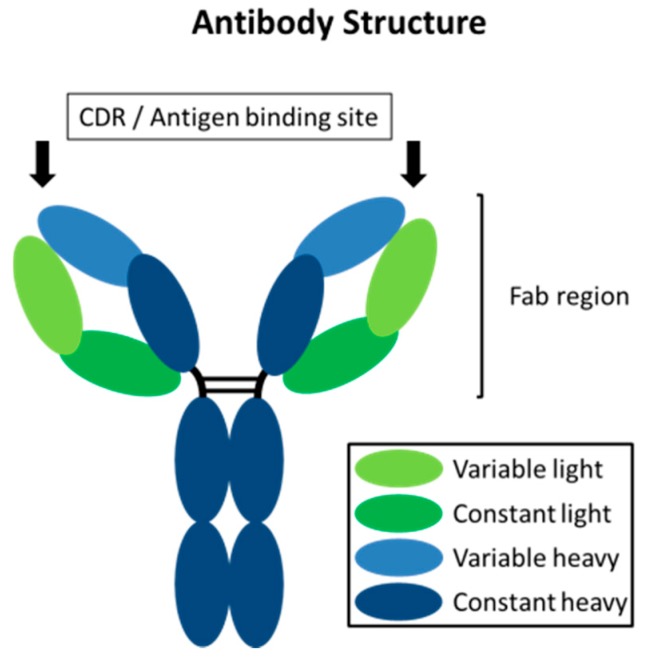
Schematic representation of an antibody with the heavy and light chains and the antigen binding site, with the complementarity-determining regions (CDRs) within the Fab region.

Antibodies represent a means of achieving a high degree of selectivity towards disease-causing biological targets. They are involved in the immune response of an organism to neutralize pathogens, such as viruses and pathogenic bacteria, through binding to their surface with the antigen binding site within the Fab region, as displayed in Figure 1 [6,7,8].

Compared to small molecule drugs, the antibodies show some beneficial characteristics, such as high target selectivity which includes no hERG related toxicity. Further, the biological drugs display a longer half-life of days or weeks which enables an intermittent dosing. Metabolism of the antibody drugs results in degradation products like peptides or amino acids, which can be recycled by the body. These properties make inhibitory antibodies a promising tool for the inhibition of enzymes. Nevertheless, safety issues and side-effects were also reported for inhibitory antibodies. Antibodies have unique toxicities that differ from those of traditional chemotherapeutics. Safety problems often relate to immunomodulation and infection and manifestation can range from local skin reaction at the injection site, to acute anaphylaxis and systemic inflammatory response syndrome, which can be fatal. Other adverse events include cardiovascular and pulmonary problems, and cancer, for example, that were reported to occur after administration of antibody drugs, forcing the discontinuation of the therapies [9,10]. Numerous targets inhibitory antibodies already exist that proved to be effective in vitro and in vivo [11,12,13,14,15,16,17]. On the matrix metalloproteinase platform, the gelatinase B (MMP-9) and the membrane-type matrix metalloproteinase-1 (MMP-14), both validated targets in pathogenic events, can be blocked selectively with inhibitory antibodies [18,19,20,21,22,23,24,25,26,27,28,29,30,31,32,33,34]. This emerging field of interfering with single MMPs through inhibitory antibodies provides new options on the road to finally tackle MMP related diseases and get a corresponding drug on the market.

## 2. Matrix Metalloproteinases

Matrix metalloproteinases (MMPs) are proteolytic enzymes found in healthy organisms where they are involved in physiological processes like angiogenesis, wound healing, and embryonic development, as well as in regulating inflammatory processes [35,36,37,38,39,40,41,42]. Their activity is strongly regulated by the tissue inhibitors of metalloproteinases (TIMPs) [43]. An imbalance in this system, leading to increased MMP activity, is associated with a multitude of severe diseases like cancer, arthritis, chronic obstructive pulmonary disease (COPD), or sepsis [36,44,45,46,47,48,49,50,51,52,53,54,55,56,57,58]. The general structure of the MMP family members is very similar and incorporates up to three Ca^2+^ ions and a Zn^2+^ ion as structural elements. A second Zn^2+^ ion within the catalytic domain, which is complexed by three histidines, forms the active center where protein hydrolysis takes place, using a water molecule. As MMPs are able to decompose proteins, including themselves through autoproteolysis, they are expressed as inactive zymogens where the catalytic center is complexed with a single cysteine residue of the propeptide domain. To obtain catalytic activity, the complex has to be dissociated through a “cysteine switch”, enabling a water molecule to coordinate to the catalytic zinc [59].

### 2.1. Classes

MMPs can be divided into two main groups according to whether they are secreted to the extracellular matrix (ECM) or anchored to the cell surface. Further, they are classified according to their preferential substrate: The collagenases (MMP-1, -8, -13, -18), gelatinases (MMP-2, -9), stromelysins (MMP-3, -10, -11), MT-MMPs (MMP-14, -15, -16, -17, -24, -25), and others (MMP-7, -12, -19, -20, -21, -22, -23) together have the ability to decompose all components embodied in the extracellular matrix.

A challenge for pharmaceuticals targeting MMP-related diseases lies in the high redundancy within the MMP network. If one MMP is selectively inhibited or knocked-out in an animal model, other MMPs might step into action and compensate for the lack in function. This can be traced back to the fact that each MMP in the network can hydrolyze multiple substrates and different substrates can be cleaved by various MMPs. In a MMP-13 knock-out mice model, for example, enhanced MMP-8 expression was observed during wound healing [60]. An increase in MMP-9 expression was observed in a MMP-8 deficient wound repair model [61]. This redundancy might be a possible explanation for the unsatisfying outcome in clinical trials with selective MMP inhibitors so far. 

### 2.2. Mechanism of Cleavage

The mechanism by which metalloenzymes degrade their substrates has been examined for decades for several enzyme classes [62,63,64]. Evans and Cravatt have extensively reviewed a wide spectrum of enzyme families and their mechanism of action [65]. A widely accepted mechanism was postulated by Matthews for thermolysin and related zinc peptidases [66].

**Figure 2:**
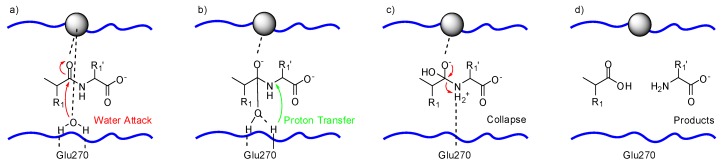
Proposed mechanism for the catalyzed hydrolysis of peptide bonds by carboxypeptidase A: (**a**) nucleophilic attack of the water molecule, (**b**) proton transfer to the substrate, (**c**) collapse of the intermediate results in (**d**) the formation of the products, zinc (II) ion depicted as gray sphere; based on Matthews [66].

The incoming substrate drives the catalytic, zinc-bound water towards Glu270. The water oxygen ligated to the zinc ion, as shown in Figure 2a, has enhanced nucleophilicity by having both its protons hydrogen bonded to Glu270. Glu270 and zinc promote the attack of water on the carbonyl carbon of the substrate, as shown in Figure 2b. Glu270 accepts a proton from the water molecule and immediately shuttles it to the amide nitrogen at the scissile peptide bond of the substrate, as depicted in Figure 2c. The collapse of the intermediate, displayed in Figure 2d, delivers the hydrolyzed products.

### 2.3. Structural Studies

**Figure 3:**
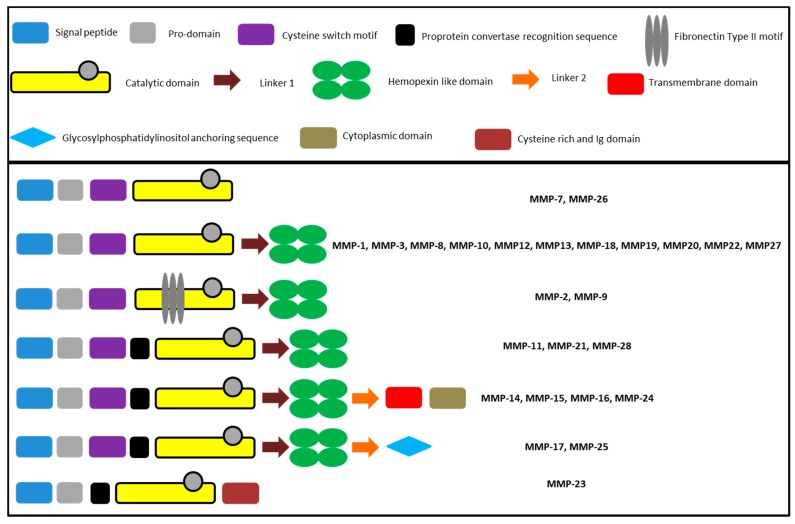
Composition of MMPs according to Nagase et al. [43].

The general composition of matrix metalloproteinases comprises conserved units as well as structural features only found in a fraction of MMPs. All MMPs share a pro-peptide domain of approximately 80 amino acid residues in length, incorporating a PRCGXPD motif interacting with the active site zinc ion to block the catalytic center in the inactive pro-form of the enzyme. The removal of the interacting cysteine, referred to as the cysteine-switch, has to occur for the activation of the proteolytic activity [59]. MMP-2 and MMP-9 possess three fibronectin type II domains for the interaction with the collagen [67] and MMP-23 has a cysteine rich domain [68]. With the exception of MMP-7, -23, and -26, all isoforms possess a C-terminal hemopexin-like domain composed of about 190 amino acids [69]. Figure 3 summarizes the schematic composition of the MMPs.

Within the catalytic domain of about 170 amino acids, the matrix metalloproteinases possess a zinc-binding motif HEXXHXXGXXH as well as a conserved methionine, forming a “Met-turn”, a structural feature which is shared with other members of the metzincins [70]. The zinc-binding motif complexes three of the four coordination sites of the catalytic zinc, leaving the remaining position free for a water molecule in the active form or a cysteine in the zymogen prior to the cleavage. In addition to the catalytic zinc, MMPs incorporate a second zinc ion and up to three calcium ions for the stability of the active enzymatic conformation [71,72].

**Figure 4:**
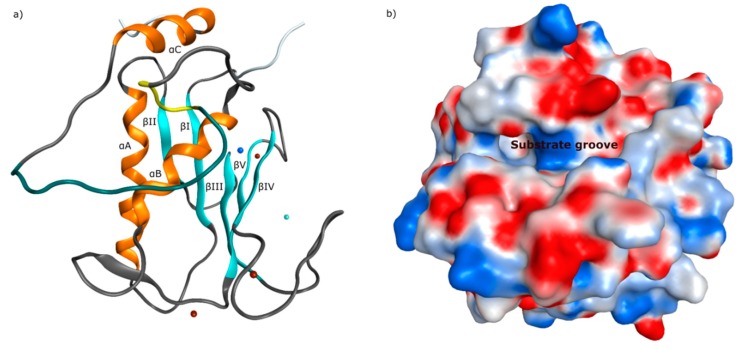
(**a**) Ribbon structure of catMMP-13 (PDB ID: 2OW9) [73] showing five beta-sheets and three alpha-helices (catalytic zinc(II) ion as blue and structural zinc(II) ion as cyan sphere, calcium(II) ions as brown spheres), (**b**) electrostatic surface of catMMP-13 with the substrate recognizing groove.

The tertiary structure of the catalytic domain is similar in all matrix metalloproteinases. It contains three alpha-helices (αA–αC) and five beta-sheets (βI–βV), as outlined in Figure 4a. The arrangement of the structural features results in an active conformation of similar shape for all members of the MMP family. By topological consideration, it is visible that the catalytic domain forms a linear cleft close to the catalytic zinc, where the scissile substrate inserts, as visible in Figure 4b. In an angle of approximately ninety degree from the substrate groove, a channel is located next to the catalytic zinc (II) ion, where the P1’ portion of the particular substrate is recognized. The S1’ channel alters in size and shape within the enzyme family, making it a valuable target for the design of selective MMP inhibitors.

#### S1’ Pocket Comparison

**Figure 5:**
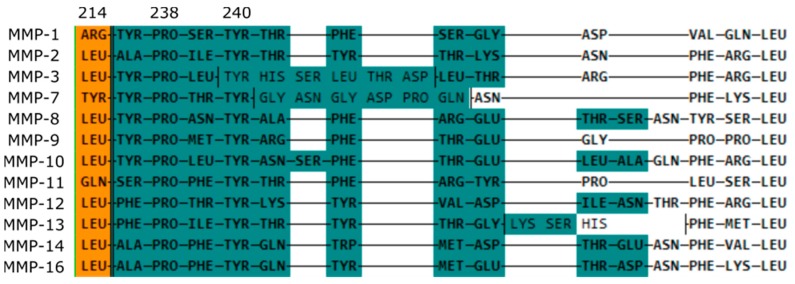
Sequence alignment of the S1’ pocket-forming residues of the MMPs, for which crystallographic data is available. (PDB IDs: MMP-1 1HFC [74], MMP-2 1QIB [75], MMP-3 1HY7 [76], MMP-7 2Y6D [77], MMP-8 1I76 [78], MMP-9 4XCT [79], MMP-10 3V96 [80], MMP-11 1HV5 [81], MMP-12 1Y93 [82], MMP-13 5B5O [83], MMP-14 3MA2 [84], MMP-16 1RM8 [85]).

Within the catalytic domain, all MMPs share a structural feature, which is important for the recognition of the preferred substrate. The so called S1’ pocket is located in close proximity to the catalytic zinc (II) ion and it varies in length and shape among the different MMPs (Figure 5).

According to this variety, the MMPs can be grouped into members with a small (MMP-1, MMP-7, MMP-11, and MMP-20), medium (MMP-2, MMP-8, MMP-9, MMP-12, MMP-14, and MMP-16), and large (MMP-3, MMP-10, and MMP-13) S1’ pocket. Fabre et al. have reviewed the dynamics of this structural entity for the design of selective small molecule MMP inhibitors [86]. The green marked amino acids in Figure 5 make up the specificity loop in close proximity to the catalytic zinc(II) ion and the amino acid in position 214 (orange, numbering according to full MMP-3) is located within the S1’ channel. For most MMP family members, this position is occupied by a Leucine, but MMP-1, MMP-7, and MMP-11 possess an Arginine, a Tyrosine, and a Glutamine at this site. Given the steric demand of the sidechains, the available space within the selectivity pocket is limited, resulting in shorter channels for those members. The different sequences in various MMP S1’ channels and the resulting difference in size, shape, and surface property results in substrate specific recognition and simultaneously gives an opportunity for the design of tailor-made selective inhibitors against a certain MMP.

## 3. MMP Inhibition

**Figure 6:**
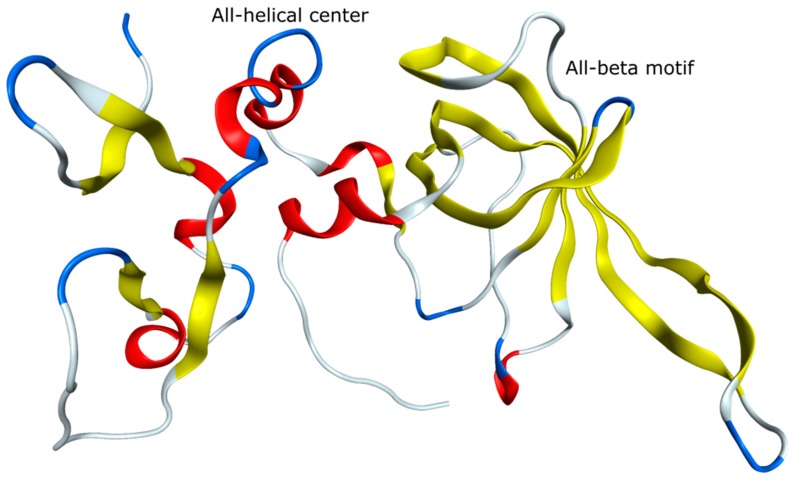
Structure of TIMP-2 (PDB 1BR9) [93].

In healthy organisms, MMP activity is regulated by endogenous TIMPs that are natural inhibitors of MMPs as well as of a disintegrin and metalloproteinases (ADAMs) and ADAMs with thrombospondin motifs (ADAMTs) [87,88,89]. High TIMP levels lead to ECM accumulation due to inhibition of the degradation processes, whereas low TIMP activity results in elevated proteolysis. All four members of the TIMP family (TIMP-1, -2, -3, -4) inhibit the according enzyme by formation of stoichiometric complexes [90,91]. Structurally, they consist of two distinct domains, an N-terminus of ~125 amino acid residues and a C-terminus of ~65 amino acids in length [90]. Conformations characterized by an elongated contiguous wedge consisting of the N-terminal segment, an all-β-structure motif, and an all-helical center (Figure 6) are inherent throughout the TIMPs [90,91]. The function inhibiting complexes of TIMPs with the MMPs are stabilized by interactions of the C-terminal domains of the TIMPs with the hemopexin-like domain in most MMPs, and by interactions of the TIMPs N-terminal domain with the zinc-ion within the catalytic domain of the MMPs [88,89,90,91]. Very recently, the Riedl group analyzed such a MMP-TIMP complex and used it for the de novo design of highly potent and selective MMP inhibitors [92].

As an imbalance between MMPs and TIMPs plays a critical role in a variety of diseases, the re-establishment of a balanced MMP activity profile delivers an approach for the development of therapeutics [94,95,96,97,98]. Modulation of MMP activity can be achieved with TIMP analogues as described by Arkadash et al. where they modified the non-specific N-TIMP-2 (N-terminal domain of TIMP-2) to obtain a mutant that is highly potent against MMP-14 with a K_i_ of 0.9 pM and up to 16’000-fold selectivity over other MMPs [99]. Further, peptidomimetics with structural similarity to TIMPs showed inhibition of selected MMPs with high potency and selectivity. Gall et al. described a cyclic TIMP peptidomimetic for the inhibition of MMP-2, MMP-9, and MMP-13 (IC_50_ of 170 nM, 140 nM and 21 nM, respectively) [92]. Numerous small molecule inhibitors have been developed in the past for the modulation of MMPs [1,100]. Early representatives inhibited all MMPs without discriminating between the subtypes which led to undesired side effects and made the development of selective inhibitors necessary. At present, there exist many small molecule inhibitors with a narrow spectrum or even selectivity for one specific MMP over all other family members. Nevertheless, so far, no small molecule modulator of MMPs has emerged to the market as all failed in clinical trials due to unsatisfying performance.

As the enzyme class is still an intriguing target in pharmaceutical research, novel strategies are under investigation to modulate the MMP microenvironment. One technique that has already delivered promising results is the engineering of inhibitory antibodies tailored to only block a single MMP.

## 4. Antibodies for MMP Inhibition

In addition to the aforementioned natural and synthetically obtained small molecule inhibitors, there exists a third class of downregulating modulators of matrix metalloproteinases. Anti-MMP antibodies can be designed and engineered for the selective inhibition of single MMP isoforms. Such inhibitory antibodies have already been developed successfully for gelatinase B (MMP-9) as well as for MT1-MMP (MMP-14) [23,24,28,30,101,102].

### 4.1. Anti-MMP-9 Antibodies

By producing human MMP-9 and using it as an antigen in a mouse, Paemen et al. prepared mouse monoclonal antibodies (mAb) against gelatinase B [18]. Out of five selected antibodies with variable epitope specificity and inhibitory activity, REGA-3G12 displayed superior properties in terms of binding to both biotinylated as well as natural MMP-9, with a K_d_ of 2.1 nM. As none of the tested antibodies showed cross-reactivity to the closely related MMP-2, they can be considered as selective MMP-9 inhibitors. REGA-3G12 inhibited catalysis by gelatinase B in a degradation assay of nasal septum type II gelatin and it inhibited the degradation of biotinylated gelatin in a solution assay [18]. Modifications of REGA-3G12 towards single-chain variable fragments (scFv) resulted in monomeric forms that were all less active than the template antibody [21]. Further investigation on the mode of inhibition revealed that REGA-3G12 interacts solely with the catalytic domain of MMP-9 and not with the fibronectin repeats or the hemopexin domain (PDB not available). It recognizes the amino terminal part and does not bind at the catalytic zinc-containing center. Those findings suggest that a part of the catalytic domain (not the zinc-binding domain) can serve as target for the development of selective inhibitors [22].

Recently, Sela-Passwell et al. have produced inhibitory antibodies with similar binding mechanisms against gelatinases as the endogenous TIMPs [103]. Seeking an antibody that targets the zinc(II) ion within the catalytic site, they have used a strategy in which mice were immunized with a synthetic molecule mimicking the zinc–histidine complex, conserved in metalloenzymes. This set-up yielded the function-blocking monoclonal antibodies SDS3 and SDS4 that inhibited MMP-9 and the closely related MMP-2 (K_i_ values of 1 and 0.054 μM for SDS3 and SDS4, respectively) while showing, by an order of magnitude, lower inhibition of MMP-14 and sparing MMP-1, MMP-7, and MMP-12. Surface plasmon resonance measurements of the antibodies showed that MMP-9 binding strongly interfered in the presence of acetohydroxamic acid, suggesting a direct interaction between the inhibitory antibodies and the catalytic zinc(II) ion in MMP-9. The antibodies bind their target MMPs through protein–protein interactions with respect to the metal–protein motif, as well as to the enzyme surface. Further selectivity towards a single MMP may be achieved by classical protein engineering procedures that refine protein surface interactions between the antibody and the target enzyme [103].

Researchers at Gilead Sciences reported the development of Andecaliximab (GS-5745), a humanized monoclonal antibody, as a potent (K_d_ = 0.168 nM) and highly selective allosteric inhibitor of the gelatinase B (MMP-9) that spares MMP-1, -2, -3, -7, -8, -10, -12, -13, -14, and -16 with K_d_-values of greater than 100 nM [104]. As shown in the study, the selective inhibition of MMP-9 did not induce side effects such as musculoskeletal syndrome, as was the case for broad-spectrum MMP inhibitors. In preclinical investigations, MMP-9 inhibition reduced the disease severity in a mouse model of ulcerative colitis and it decreased tumor growth and metastases incidence in a surgical orthotopic xenograft model of colorectal carcinoma. Further, it was shown that inhibition of either tumor, or stroma-derived MMP-9, was sufficient to reduce primary tumor growth [104].

**Figure 7:**
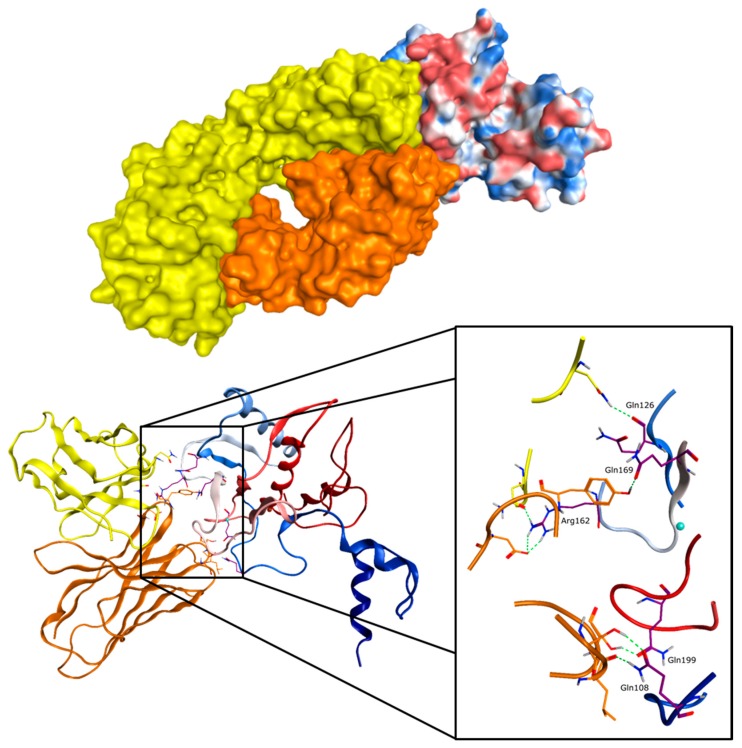
Crystal structure of GS-5745 Fab’ fragment complexed to MMP-9 and a close-up of the interaction site with the interacting side chains of MMP-9 (PDB ID: 5TH9 [19], GS-5745 Fab fragments in yellow and orange, and MMP-9 with electrostatic color surface).

Structural investigation revealed that GS-5745 binds MMP-9 distal to the active site, near the junction between the prodomain and the catalytic domain, and inhibits MMP-9 by two mechanisms: Binding to pro-MMP-9 prevents MMP-9 activation, whereas binding to active MMP-9 allosterically inhibits activity [19]. For the recognition of MMP-9-pro-cat, an antibody fragment (Fab’) of GS-5745 was generated to facilitate structure determination. The fragment interacts with approximately 2130 Å^2^ of the enzyme surface and the formed complex is stabilized by hydrogen bonds with the glutamines 108, 126, 169, and 199 of MMP-9, one salt bridge to Arg162 of MMP-9, and hydrophobic interactions. The nearest residue of GS-5745 is in approximately 17 Å distance to the active site zinc(II) ion so the antibody does not occlude the catalytic site, which is in consistency with the proposed allosteric mode of inhibition (Figure 7) [19].

The therapeutic promise of an anti-MMP-9 antibody led to clinical trials with GS-5745. Andecaliximab was examined with mFOLFOX6 (combination chemotherapy regimen that includes the drugs leucovorin calcium, 5-fluorouracil, and oxaliplatin) in a phase Ib study with gastric and gastroesophageal junction adenocarcinoma, and demonstrated encouraging beneficial effects without added toxicity [105]. Decreased free MMP-9 suggested inhibition of MMP-9 enzymatic activity by GS-5745. A phase III study (NCT02545504) currently investigates the efficacy and safety of GS-5745 combined with mFOLFOX6 in subjects with untreated gastric and gastroesophageal junction adenocarcinoma [106].

For the treatment of inflammatory bowel disease complications with fibrosis, Goffin et al. investigated therapeutic efficacy of anti-MMP-9 antibodies [20]. It has been described that upregulation of MMP-9 expression is observed in inflamed mucosa or serum of patients. The researchers generated the anti-MMP-9 antibody CALY-001 and evaluated its efficacy in a mouse model of intestinal fibrosis in comparison to AB-0046-h4, a potent and selective allosteric mouse monoclonal antibody against MMP-9 that reduced disease severity in a dextran sodium sulfate-induced mouse model of ulcerative colitis [104]. CLAY-001 is an inhibitor of MMP-9 enzymatic activity, whereas AB-0046-h4 inhibits the activation of pro-MMP-9 which leads to decreased MMP-9 activity. Since both antibodies reduced the disease severity in a mouse model of intestinal inflammation, they were assessed for antifibrotic activity in an intestinal fibrosis model where they showed that inhibiting MMP-9 activity significantly restrains fibrogenesis [20].

### 4.2. Anti-MMP-14 Antibodies

It has been proposed that MMP-14 plays a crucial role in pathological systems, such as tumor growth, invasion, and neovascularization. It is able to cleave matrix proteins and it activates proMMP-2, which leads to an amplification of pericellular proteolytic activity. Using phage display technology, Devy et al. have discovered the highly selective human MMP-14 inhibitory antibody DX-2400 [102]. It efficiently blocks proMMP-2 activation and displays anti-invasive activity in vitro. As MMP-14 is involved in physiologic and pathologic angiogenesis, its inhibition by DX-2400 exhibited reduced angiogenesis through inhibition of VEGF-driven cell invasion and proMMP-2 activation. In in vivo studies, the single agent or combination application of DX-2400 markedly affected growth of MDA-MB-231 and BT-474 tumor cell lines. Pilot toxicology studies did not reveal any clinical or histologic findings, including abnormalities of the joints, offering a promise that DX-2400 will be tolerated in the clinic [102]. Further in vivo studies of murine breast tumor models showed that MMP-14 blockade by DX-2400 decreased immunosuppressive TGFβ, polarized macrophages to an antitumor phenotype, increased inducible nitric oxide synthase, and improved tumor perfusion, resulting in reduced primary tumor growth and enhanced response to radiation therapy, especially in high MMP-14 expressing tumors [107].

In 2013, Ingvarsen and Porse et al. described the monoclonal antibody 9E8 that selectively inhibits a single function of the multifunctional MMP-14 [27]. The antibody completely blocks the ability of MMP-14 to activate proMMP-2 without interfering with its proteolytic activity. Employing this antibody, they could show that the MMP-14 catalyzed activation of proMMP-2 is involved in the outgrow of cultured lymphatic endothelial cells in a collagen matrix in vitro, as well as in lymphatic vessel sprouting ex vivo [27]. As the antibody does not interact with other members of the membrane type MMP family, Shiryaev et al. examined the mechanism of this selectivity using mutagenesis, binding and activity assays, and in silico modeling. They demonstrated that the 9E8 antibody recognizes the membrane type loop structure distant from the active site [33]. 

**Figure 8:**
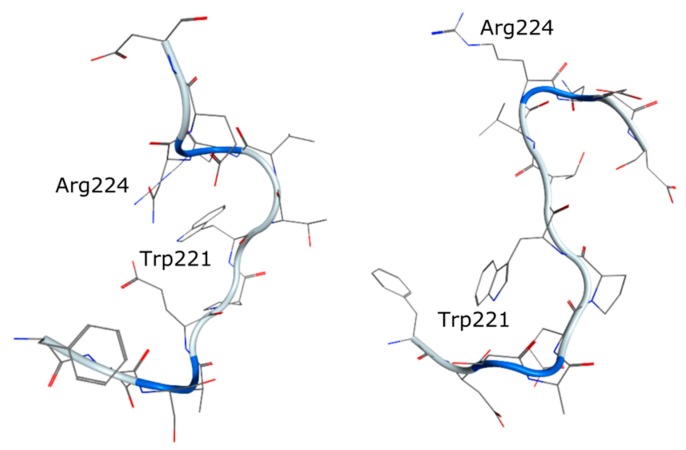
Structural comparison of the loop residues 216–227 in MMP-14. Left: bound to LEM-2/15 (PDB ID: 4P3D) [34] and right: the loop conformation of MMP-14 bound to TIMP-1 (PDB ID: 3MA2) [84]. The observed conformational change can explain the inhibition due to allosteric modification of the enzyme structure.

In comparison to the above mentioned antibodies (DX-2400 and 9E8) which inhibit the proMMP-2 activating function of MMP-14 while leaving the proteolytic function untouched, Udi et al. published LEM-2/15 that inhibits the collagenolytic function of MMP-14 selectively [34]. It inhibits the cleavage of the native substrates, collagen type I and gelatin. As within the MMP family, the members vary in terms of the nature of the V-B loop residues, and the LEM-2/15 antibody was generated by immunizing mice with a cyclic peptide that mimics the corresponding sequence in MMP-14. For easier handling, they generated a minimized antibody fragment that inhibited MMP-14 catalytic domain with an IC_50_ of 45 nM. Kinetic analyses for the determination of the inhibition type showed a noncompetitive inhibition pattern and the finding that Fab LEM-2/15 did not compete with the substrate binding at the catalytic center indicated that Fab LEM-2/15 modulates the catalytic activity allosterically by binding at the V-B loop away from the active site. Structural comparison of the free Fab fragment and complexed to a segment of MMP-14, as in Figure 8, suggests a major conformational change of the protease while binding to LEM-2/15.

This conformational change leads to narrowing of the substrate-binding cleft, which delivers an explanation for the inhibition of the collagenolytic function as it prevents substrate binding. As in some stages of cancer progression MMP-2 represents an anti-target, and activation of proMMP-2 is beneficial, the collagenolytic activity of MMP-14 remains a target to address and LEM-2/15 could serve as a potential novel therapeutic [34].

Nam et al. synthesized a human Fab antibody library in which they varied the complementarity determining region (CDR)-H3 by implementing an extended segment [108]. The strategy evoked from analyzing known camelid inhibitory antibodies, which contain long and convex-shaped paratopes that penetrate into the catalytic cleft for the inhibition of the enzymatic activity. In the study, 23- to 27-residues long CDR-H3s were synthesized for the putative formation of the convex-shaped paratopes. The constructed CDR-H3s, in contrast to the normal length CDR-H3s, displayed inhibition of MMP-14. Inhibitory antibody Fab 3A2 with a 27-residue long CDR-H3 modification proved highly affine to the target enzyme, with a K_d_ of 4.85 nM that inhibited MMP-14 with an IC_50_ of 9.7 nM, which is in the same order of magnitude as the endogenous TIMP-2 (5.1 nM) and the hydroxamate based non selective inhibitor GM6001 (2.1 nM). It was suggested that Fab 3A2 targets the S1’ pocket within MMP-14 and competes with TIMP-2 and the substrate without reaching out to the catalytic zinc(II) ion [32,108]. As Fab 3A2 can be cleaved by high concentrations of MMP-14, mutations were introduced that vary in the positions adjacent to the cleavage site, which led to more stable mutants with prolonged half-life and high potency [109,110]. In a syngeneic mouse breast cancer model with fast and spontaneous metastasis, IgG 3A2 displayed significant impact on tumor growth as well as metastatic spread and proved its therapeutic potential [25]. The strategy of synthesizing convex paratope antibody libraries might also facilitate the design of inhibitory antibodies for other enzymes in the future [108]. In the same group, they have obtained a highly affine (EC_50_ = 8.3 nM) and selective inhibitory antibody for the inhibition of MMP-14 by grafting a cyclic peptide motif GACFSIAHECGA (Peptide G), previously identified as a selective inhibitor [111], into the complementarity determining region (CDR) of an antibody scaffold [31]. The Fab 1F8 inhibited MMP-14 with a K_i_-value of 110 nM, which is, by three orders of magnitude, more potent than the Peptide G with 150 μM.

Recently, Ling et al. disclosed IgG 3369 and Fab 3369 deriving from screening a phage displayed synthetic humanized Fab library [29]. Fab 3369 is with an IC_50_ of 62 nM against MMP-14 similarly potent as the hydroxamate based broad-spectrum inhibitors and was therefore used as a lead inhibitory antibody for further investigations. In an in vitro assay with MDA-MB-231 cells, Fab 3369 was able to block endogenous MMP-14 expressed on the cell surface, and to inhibit extracellular matrix degradation and triple-negative breast cancer (TNBC) cell invasion. For continuing experiments, they employed the clone as a human IgG1 and tested it in mammary orthotopic xenograft assays using MDA-MB-231 cells injected in mice lacking NK (natural killer), B, and T cells. Mice treated with IgG 3369 showed a significant reduction in tumor growth and reduced tumor mass at the endpoint. The IgG and vector control groups revealed more lung metastases compared to the IgG 3369 treated or the MMP-14 knock-down group. The blockade of MMP-14 by IgG 3369 also disrupts the hypoxic TNBC tumor microenvironment leading to tumors with reduced density, and it impairs tumor progression and metastasis in a syngeneic breast cancer model.

## 5. Conclusions

This review highlights the emerging field of designing inhibitory antibodies for the selective inhibition of matrix metalloproteinases. The MMP family has been an intriguing target class for pharmaceutical researchers in academia and in industry for almost three decades. The first peak of interest resulted in a flood of small molecule inhibitors that did not discriminate between the individual members, resulting in failed clinical trials due to intolerable side effects. Further development gave rise to more selective small molecule inhibitors that inhibited only one member or a small subset of the target class, but until today, none of them have reached the market. Major setbacks such as toxicity, lack of selectivity, or therapeutic efficacy have impeded their advancement.

Therefore, alternatives are needed and the development of inhibitory antibodies that specifically target one single MMP hold great promise as future therapeutics. Although this field is still very young and there will certainly be many more inhibitory antibodies against individual MMPs in the future, outstanding results have already been achieved. The potent and highly selective antiMMP-9 monoclonal antibody GS-5747 displayed positive effects in the treatment of ulcerative colitis and gastric cancer. A combination study of GS-5745 with mFOLFOX6 currently investigates the efficacy and safety in patients with untreated gastric and gastroesophageal junction adenocarcinoma in a phase III clinical trial. With the latest successes in the field, the probability increased significantly that a drug will be developed that targets this enzyme family and enables clinical efficacy in the treatment of MMP-related diseases.
